# Validation of an integrative mathematical model of dehydration and rehydration in virtual humans

**DOI:** 10.14814/phy2.13015

**Published:** 2016-11-29

**Authors:** W. Andrew Pruett, John S. Clemmer, Robert L. Hester

**Affiliations:** ^1^ Department of PhysiologyCenter for Computational MedicineUniversity of Mississippi Medical CenterJacksonMississippi

**Keywords:** Dehydration, integrative physiology, mathematical model, vasopressin

## Abstract

Water homeostasis is one of the body's most critical tasks. Physical challenges to the body, including exercise and surgery, almost always coordinate with some change in water handling reflecting the changing needs of the body. Vasopressin is the most important hormone that contributes to short‐term water homeostasis. By manipulating vascular tone and regulating water reabsorption in the collecting duct of the kidneys, vasopressin can mediate the retention or loss of fluids quickly. In this study, we validated HumMod, an integrative mathematical model of human physiology, against six different challenges to water homeostasis with special attention to the secretion of vasopressin and maintenance of electrolyte balance. The studies chosen were performed in normal men and women, and represent a broad spectrum of perturbations. HumMod successfully replicated the experimental results, remaining within 1 standard deviation of the experimental means in 138 of 161 measurements. Only three measurements lay outside of the second standard deviation. Observations were made on serum osmolarity, serum vasopressin concentration, serum sodium concentration, urine osmolarity, serum protein concentration, hematocrit, and cumulative water intake following dehydration. This validation suggests that HumMod can be used to understand water homeostasis under a variety of conditions.

## Introduction

Water homeostasis is an important physiological task to study: hospital readmissions due to dehydration are critical sources of cost following bariatric surgery, ileostomy, and pancreaticoduodenectomy (Whipple), as well as in end‐of‐life care. For example, a readmission increases the 180 day cost of bariatric surgery by $180,000 (Encinosa et al. 2006). Dehydration was reported at 2% of readmissions from roux‐en‐Y gastric bypass and 11% of adjustable gastric banding surgeries (Dorman et al. 2012). For ileostomy, 43% of the 16% readmission rate is attributable to dehydration (Messaris et al. 2012). For pancreaticoduodenectomy, the readmission rate is 28%, with 16% due to dehydration (Hari and Rosenzweig 2012). Additionally, patient quality of life is implicitly reduced by readmission, so ethical considerations lend additional import to better understanding dehydration and rehydration.

Water homeostasis in humans, even in the acute setting, is managed by multiple control systems that interact with one another, but these efforts are led by AVP (vasopressin or antidiuretic hormone, ADH). Additional controlling factors include the baroreflexes (carotid and cardiac, which also induce AVP secretion), the autonomic nervous system, capillary filtration and lymph flow, AVP, and atrial natriuretic peptide (ANP), as well as the roles played by individual subcompartments of the circulatory system such as the splanchnic circulation and the large veins. It is challenging to understand all these interactions in both acute and chronic settings. One way to understand and analyze complicated physiological systems is through mathematical modeling.

Most mathematical models of physiology are intended to address a single aspect of a theory. This serves a dual purpose: it simplifies model construction, and it expedites model analysis. However, simple models do not suffice for complex physiological systems. As previously described, water homeostasis involves multiple systems at different times. Each of these systems may be modeled in a simple way, and integrating these simple models together generates a powerful tool for understanding water homeostasis in healthy and disease states.

The first integrative physiological model involving water homeostasis was that of Guyton, Coleman, and Granger (Guyton et al. 1972) which was designed to test the role of fluid volumes and the kidney in chronic blood pressure control. That model has been expanded to include 14 organ systems, modulated by an autonomic nervous system and an endocrine system, with the capacity for postural changes, exercise, multiple pharmaceutical interventions, hemorrhage, and dozens of other normo‐ and pathophysiologic conditions (Abram et al. 2007; Hester et al. 2011). A robust validation of these efforts has not been published. In this paper, we summarize the systems controlling short‐term water homeostasis in the model and attempt to validate it in the setting of dehydration and the transient response to rehydration. Here we demonstrate the capacity of our model to simulate dehydration/rehydration experiments previously published in human populations and to validate the model for further investigations into water homeostasis in man. The goal is not an exhaustive description of the model, but rather a multifaceted demonstration of the model's ability to reproduce different aspects of the human response to these types of challenges. To our knowledge, no integrative model of serum osmolarity and AVP exists.

## Methods

### Model

We utilize the integrative mathematical simulator HumMod, a well‐established interactive physiological model comprising some 8000 variables which has been developed over the past 40 years (Hester et al. 2011). HumMod's focus is on integrating all facets of cardiovascular control (neural, hormonal, and fluid‐dynamic) to produce a robust model of circulatory physiology, with auxiliary endocrine, metabolic, and respiratory models providing more details. Precursors of this model have been used in numerous studies whose intent was to provide a more detailed understanding of the physiologic mechanisms at play in common clinical conditions (Summers et al. 1997, 2007, 2009, 2011; Summers and Coleman 2002; Abram et al. 2007). The model is composed of mathematical expressions of the relationships between physiological variables based upon well‐understood cell/tissue/organ physiology. The details of model structure are beyond the scope of this article, and have been described previously (Hester et al. 2011). We will, however, summarize the elements of water homeostasis in the model. These fall into three categories: (1) water compartments and the ability of water to transfer from one compartment to another; (2) the kidney; and (3) the endocrine modulators of pressure and renal function. In these sections, a description and diagram of the model contents are given.

In HumMod, the body is divided into three general water spaces: intracellular, intravascular, and interstitial. The ratio of intracellular to extracellular volumes is mediated by the osmolarity of each space. Water flows between the interstitial and intravascular compartment via lymph flow and capillary filtration/reabsorption. Lymph flow is assumed to depend only on increases in interstitial pressure as valves prevent backflow (Swartz 2001), whereas capillary filtration/reabsorption is determined by Starling forces, the interactions between fluid and oncotic pressures. These transitions are calculated at three different levels to account for the differential effect of gravity in orthostatic challenges.

The kidney is modeled as a single nephron, whose action is multiplied by the number of available filtering nephrons. Flow through the afferent arteriole/glomerulus/efferent arteriole complex is calculated as an ohmic (resistance) system with no compliance. The afferent arteriole is the target of tubuloglomerular feedback originating from the macula densa (MD) as a function of sodium concentration leaving the loop of Henle (LH). The efferent arteriolar resistance is modulated by angiotensin II. Together, these act to buffer pressure changes to maintain adequate filtration at the glomerulus. The nephron itself is divided into proximal tubule (PT), LH, MD, distal tubule (DT), and collecting duct (CD). The reabsorption and secretion actions of these segments exceed the level of detail of this manuscript, but are included for completeness in the Appendix.

The endocrine modulators of water homeostasis are AVP, ANP, and renin/angiotensin II/aldosterone. AVP is released from the posterior pituitary gland in response to increased serum osmolarity or increased activity of the cardiac baroreceptors due to hypervolemia. The hormone is created in the hypothalamus and transported to the neurohypophysis for secretion. It acts in the CD to increase water reabsorption through regulation of the aquaporin‐2 channels. It also acts in the vasculature to increase resistance, which acutely increases systemic pressure and drives greater renal filtration and increased urine osmolarity. Its half‐life in circulation is 10–35 min (Mitra et al. 2011). Because we focus on AVP and serum osmolarity, a brief description of the renal model, the body water homeostasis model, AVP synthesis and secretion, and AVP dynamics and kinetics is given in the Appendix.

HumMod is available for academic download www.hummod.org/projects/.

### Protocols

In all cases, we selected studies from the literature with protocols that used hypo‐, iso‐, and hypertonic infusions or oral loads to perturb the water/salt balance in normal humans. Although exercise protocols have been used to study dehydration, we chose to focus on simpler protocols to reduce confounding effects from metabolism and mechanical reflexes, especially the exercise pressor effect, temperature elevations, and acute electrolyte disturbances more associated with exercise activity than hydration status. The protocols used are summarized in Table 1.

**Table 1:** Summary of experimental and simulation protocols

Protocol	Summary	Reference
A	Water load from basal state	Goldsmith et al. (1986)
B	36 h water deprivation followed by 15 mL/kg oral water	Williams et al. (1989)
C	24 h water deprivation with 80 mmol NaCl, followed by 10 mL/kg oral water	Seckl et al. (1986)
D	24 h water deprivation with 80 mmol NaCl, followed by 10 mL/kg oral 1.2% saline	Seckl et al. (1986)
E	5% saline infusion for 2 h at 0.7 mL/kg, followed by ad libidum oral water	Thompson et al. (1987)
F	5% saline infusion for 2 h at 0.7 mL/kg followed by no water	Thompson et al. (1987)

The first study we simulated (Protocol A) investigated the suppression of AVP following an oral water load (Goldsmith et al. 1986). Following determination of baseline serum osmolarity and AVP, an oral water load of 20 mL/kg over 20 min was given and the same measurements were repeated an hour later.

The second study (Protocol B) tested the effects of drinking volume on AVP and ANP in humans (Williams et al. 1989). Normal volunteers were assessed at baseline, after 36 h of water deprivation, and at 5, 10, 15, 30, and 60 min after different interventions. The interventions included oral intake of water, either drinking 15 mL/kg in 3.5 min (protocol DH 36H + oral H2O 15), or 1 mL/kg in 3 min (protocol DH 36H + oral H2O 1). Water deprivation was accomplished by stopping all water intake for 36 h. This protocol was replicated in HumMod based on an initial virtual body weight of 74 kg before the experiment began; this value was used to generate the total amounts of water ingested. The intake was specified as a constant rate.

The third (C) and fourth (D) protocols we simulated were designed to test the differential effects of either water or hypertonic saline on suppressing AVP secretion (Seckl et al. 1986). Six healthy volunteers were subjected to a 24 h dehydration (with an 80 mmol NaCl pill delivered during the twelfth hour). Following dehydration, subjects ingested 10 mL/kg of tap water (Protocol C) or 10 mL/kg oral saline (180 mmol/L, Protocol D) over the course of 2 min. Blood samples were drawn at 5, 10, 15, 30, and 60 min following administration of fluids. The protocol was replicated in HumMod, with the “pill” being an oral load consisting of 1 mL water and 80 mmol NaCl. Again, the measured weight of the HumMod individual was 74 kg, and intake during the drinking period was specified as constant rate.

The fifth (E) and sixth (F) protocols we simulated AVP responses in hypernatremic individuals (Thompson et al. 1987). Seven healthy volunteers were subjected to an overnight fast with ad libitum water. After a blood draw, subjects received a 2 h infusion of 5% NaCl solution at 0.06 mL/kg/min. After the infusion, a 15 min equilibration period was followed by 30 min of ad libitum tap water (Protocol E) or nothing (Protocol F). Measurements were taken every 30 min following the initiation of saline infusion. The protocol was replicated in HumMod, with “overnight fast” being defined as 10 h with no food intake, and ad lib. water defined by the model's thirst functions.

### Analysis

The results between studies are compared, with *Z*‐scores, the magnitude of the difference between experimental mean and HumMod results divided by the experimental standard deviation. Additionally, root mean square error is computed for each variable observed in each protocol.

## Results

**Figure 1:**
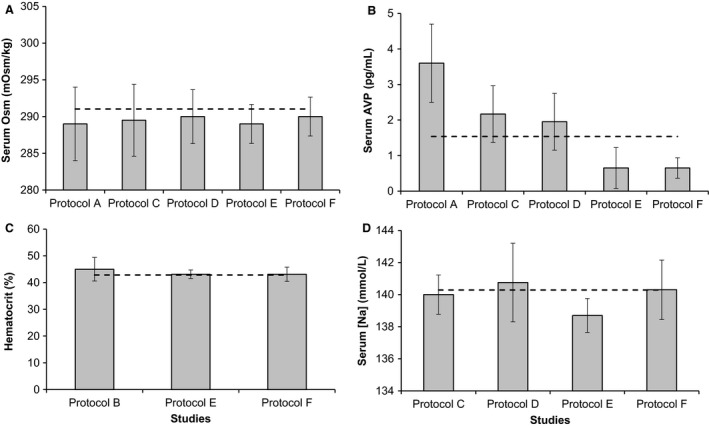
We compare the baseline variable values shared between at least three different protocols. In each case, the dashed line indicates the value of the HumMod simulation at baseline for comparison. Error bars denote standard deviation. Panel A shows baseline serum osmolarity, Panel B shows serum AVP levels, Panel C shows hematocrit, and Panel D shows plasma sodium concentration.

We used four different primary sources for data, each using different but related protocols. This was done to demonstrate the ability of the simulation, but it requires confirmation that the studies are consistent between one another. We compared each endpoint shared between at least three protocols: osmolarity (mOsm/kg) (4 studies), AVP (pg/mL) (5 studies), hematocrit (%) (3 studies), and plasma sodium concentration (mmol/L) (4 studies). These are shown in Figure 1, with the baseline HumMod value shown as a dotted line in each figure. All comparisons are made at baseline, before any insult or intervention. HumMod was within a single standard deviation of serum osmolarity and hematocrit in all cases. AVP differed significantly between the studies, although not within studies with multiple arms. HumMod was within one standard deviation of Seckl's results (Protocols C and D) (Seckl et al. 1986), which fell between Goldsmith's (Protocol A) (Goldsmith et al. 1986) relatively higher and Thompson's (Protocol E and F) relatively lower results (Thompson et al. 1987). Serum sodium was similar in HumMod to both arms of Seckl's study (C and D) and one of Thompson's “drinking” protocol (E). HumMod differed from Thompson's nondrinking protocol by less than two standard deviations (F). The nondrinking arm was significantly different than the drinking arm as well (E and F).

All protocols were divided into four different types of challenges to physiological homeostasis. The first protocol was the response to water loading from the basal state. Second was the response to dehydration over a period of 24–36 h. Third, we considered the response of the dehydrated individual to fluids following fluid restriction. Fourth was the reaction to hypertonic infusion and recovery from the same with water. These divisions will guide the remainder of the results. In all cases, the original human data are redrawn against HumMod's results for easy comparison. Additionally, HumMod's responses are shown as dashed lines, and the experimental results are shown as solid lines.

**Figure 2:**
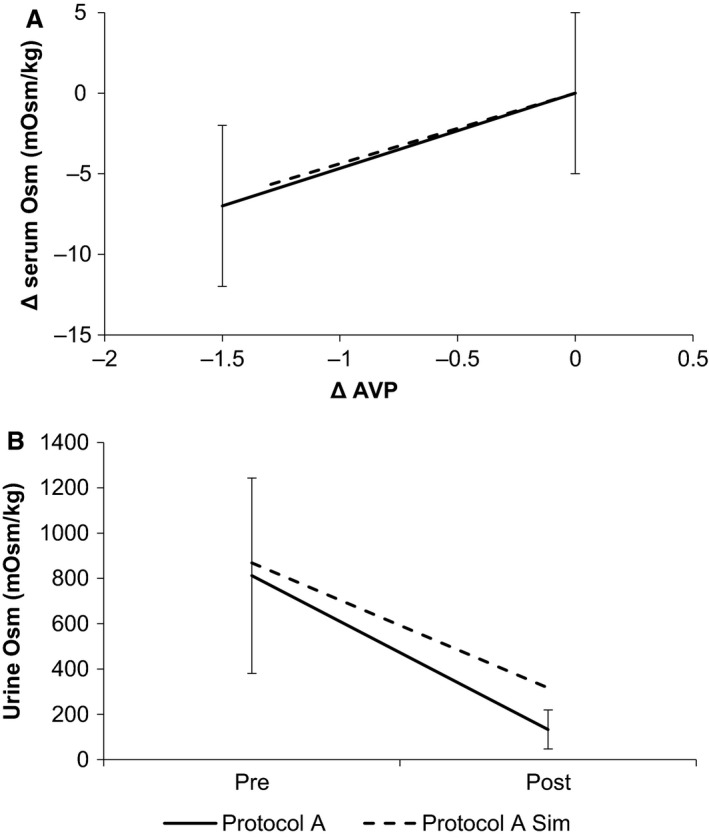
We show the HumMod simulation of Goldsmith's protocol (Protocol A), along with Goldsmith's data. Dashed lines represent simulations, whereas solid lines represent experimental data.

Goldsmith tested the response of serum osmolarity, AVP, and urine osmolarity to a water load (A). HumMod successfully replicated the osmolarity and AVP responses, staying within a single standard deviation at the pre and postexperimental data points (Fig. 2). HumMod's urine osmolarity did not fall as far as did the patients' in the study. HumMod predicted *U*_osm_ = 324 mOsm/kg, whereas the experiment showed *U*_osm_ = 133 ± 86 mOsm/kg. Sodium excretion concentration only fell by 25% (data not shown), whereas water excretion rose 20‐fold (from 0.58 to 11.89 mL/min).

**Figure 3:**
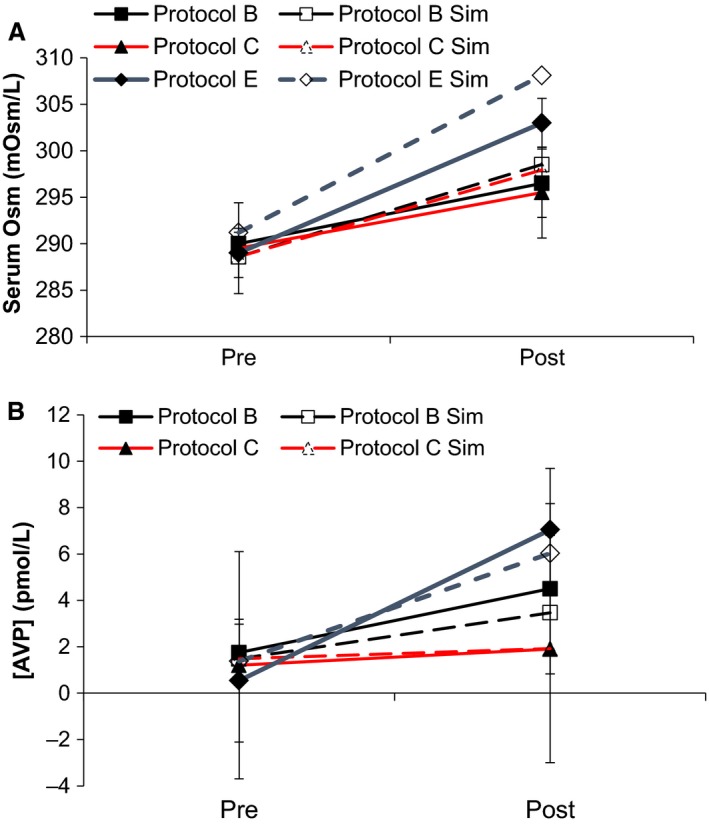
Protocols B–F began with a dehydration period. The effects of dehydration on serum AVP and osmolarity were reported at the beginning and end of dehydration, and are here compared to the simulated results from HumMod. Protocols C and D were statistically equivalent during dehydration, as were Protocols E and F, so we show only Protocols B,C, and F for clarity. Dashed lines represent simulations, whereas solid lines represent experimental data.

The studies of Williams (B), Seckl (C and D), and Thompson (E and F) were dehydration studies. Williams and Seckl used water restriction and Thompson used hypertonic saline infusion to achieve this end. They shared two measurements in common: AVP and serum osmolarity. These are shown in Figure 3 for Protocols B, C, and E, as D and F were statistically equivalent to C and E during the dehydration period. Both quantities changed significantly in the dehydration period, and HumMod showed similar changes, within one standard deviation of the experimental changes in both quantities in both Seckl's and Thompson's studies. HumMod's basal AVP was less than two standard deviations from Thompson, as was noted in Figure 1; HumMod additionally had a more significant increase in serum osmolarity than Thompson reported (<2 standard deviations). The only other observed variable that changed robustly during dehydration was serum sodium, as observed in Seckl's study. We observed a similar change, again with both endpoints being within one standard deviation of the experimental results (included in supplement).

**Figure 4:**
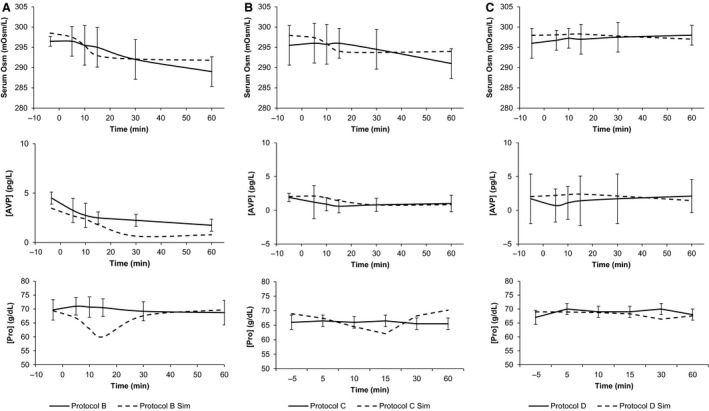
The water restriction protocols (Protocols B, C, and D) are shown along with their responses to rehydration. Serum osmolarity, AVP, and protein were measured in all three protocols and are shown for comparative purposes. Dashed lines represent simulations, whereas solid lines represent experimental data.

In both Williams' study (Protocol B) and the water arm of Seckl's study (Protocol C and D), hypotonic fluids were used to recover from water restriction. These observations yielded the acute human response to moving from a hypertonic to hypotonic state through oral intake (Fig. 4). In HumMod, serum osmolarity remained within one standard deviation of all experiments at each time point except the first in the 36 h dehydration study. The experimental serum sodium response to water loading after 24 h dehydration was similar for each measurement, as was the total serum protein response to saline loading. Williams' study (Protocol B) reported no significant changes in heart rate, and HumMod's responses were similar (remaining within a standard deviation of experimental results, data not shown). In HumMod, total serum protein dipped during recovery from water restriction (Fig. 4A), while no change was seen experimentally. Williams recorded hematocrit throughout the experiment, which we used as a proxy for the proportion of infused water that remains within the vasculature. Model hematocrit and protein changed together on a percentage basis (data not shown), indicating that increased volume caused the excessive dip. This may have been due to overly aggressive water reabsorption in the GI lumen following dehydration.

**Figure 5:**
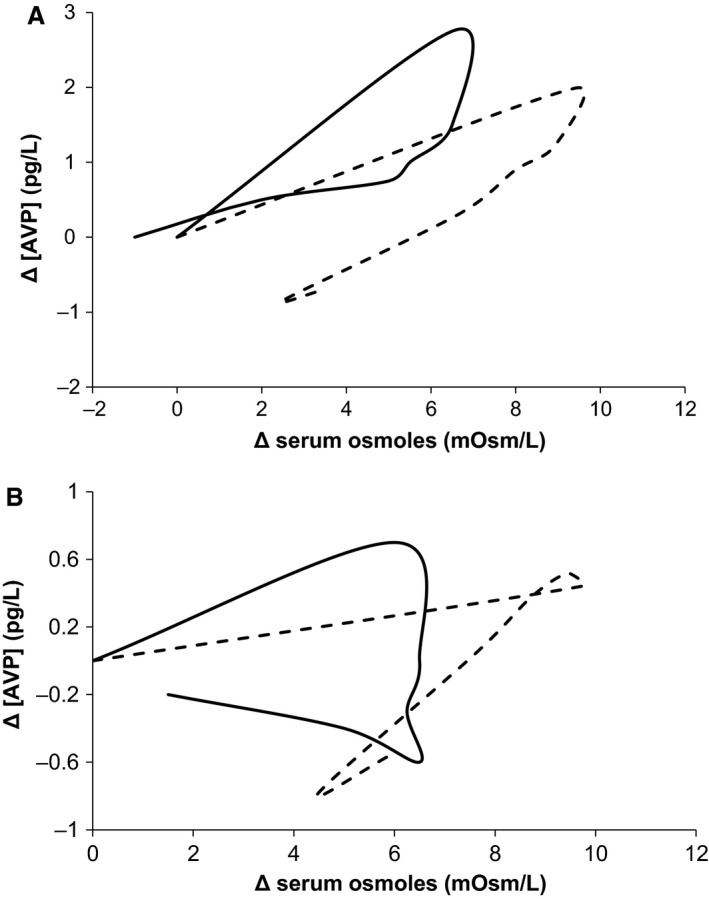
The relationship of AVP and serum osmolarity is shown in the experimental and simulation protocols B (top) and C (bottom). Dashed lines represent simulations, whereas solid lines represent experimental data.

For further confirmation of the validity of model response, the Δ[AVP] and Δ[Osm] curves for 24 and 36 h dehydration (Protocols B and C) with oral water recovery are shown in Figure 5. For clarity, error bars are dropped, and change from baseline is shown rather than absolute values. The curves are qualitatively similar, and the simulation curves lie within the first standard deviation of the experimental curves.

**Figure 6:**
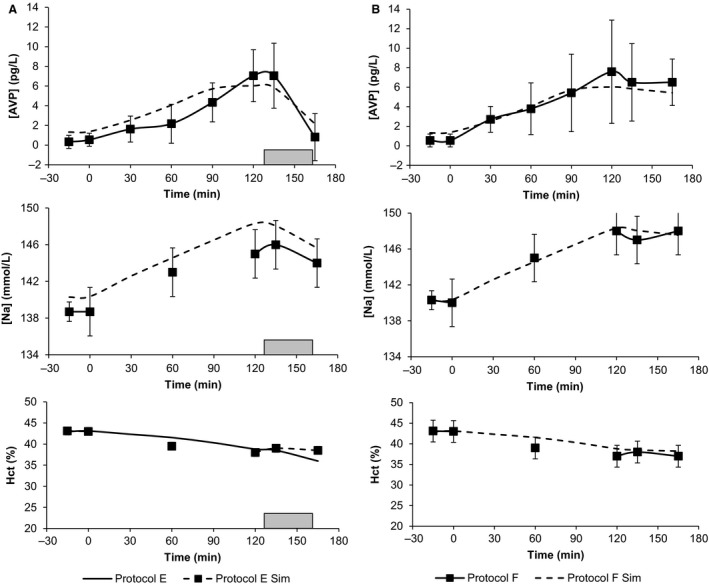
We show AVP, sodium, and hematocrit in the experimental and simulated response to hypertonic saline infusion, followed by either ad libidum water or no water (Protocols E and F). Dashed lines represent simulations, whereas solid lines represent experimental data. The gray bar in Panel A shows the water intake period.

**Figure 7:**
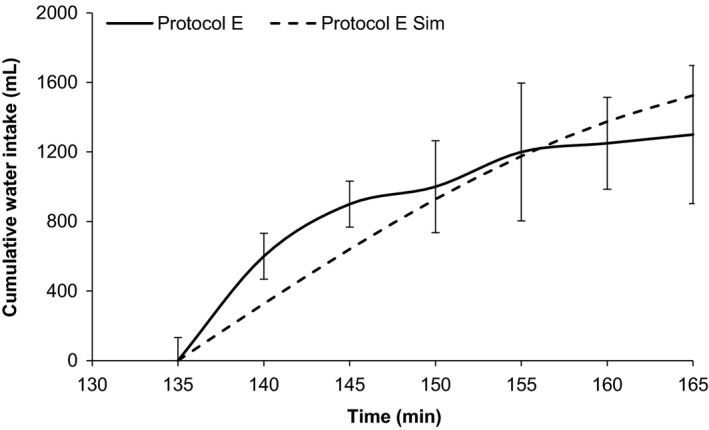
Comparison between the cumulative water intake during the ad libidum water period in Protocol E. Dashed lines represent simulations, whereas solid lines represent experimental data.

Thompson investigated the effects of ad libidum water versus no water in the response to hypervolemic hypernatremia following saline infusion (Protocols E and F). These protocols involve the patient choosing their own drinking rate and total water intake. This presents a different set of challenges to the model. Figure 6 illustrates the primary elements of the validation study. In HumMod, the change in AVP throughout the dehydration similarly reflected that seen in the actual experiments, although the absolute magnitudes were somewhat different. Recall from Figure 1 that Thompson's AVP measurements were significantly lower at baseline than that seen in the other experiments. In Protocol F, HumMod replicated the rise in serum osmolarity near perfectly; in Protocol E, the model had a persistent bias of 3 mOsm/L (data not shown). The change in serum osmolarity throughout the dehydration process did approximate the experimental record to within a standard deviation at each measurement. Serum sodium remained within 1 standard deviation at all points in Protocol F, and exceeded this bound only at the initial and final time step of the dehydration process in Protocol E. The change in hematocrit was observed as a proxy for vascular‐extravascular balance, and remained within bounds in both protocols for every measurement except the last time point of Protocol E. Again, this likely reflects water being absorbed too rapidly in the GI tract. The cumulative water intake in HumMod was similar to the experimental results (Fig. 7). At 5 and 10 min after drinking was allowed (minutes 140 and 145 of Protocol E), the model underestimated drinking rate, but the aforementioned dips in protein and hematocrit indicate that absorption was overpaced even at the reduced intake.

**Figure 8:**
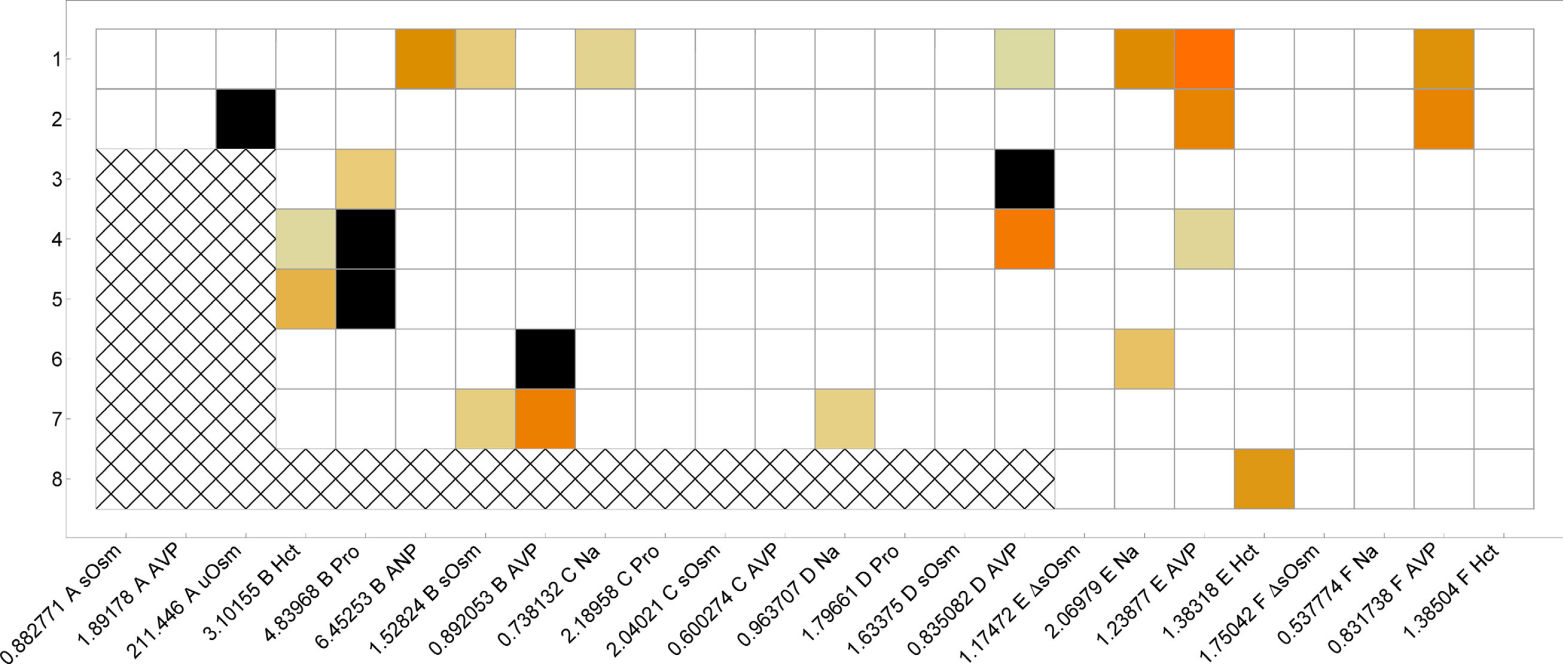
This heat map shows the *Z*‐scores of HumMod compared to every measurement discussed in this manuscript. White indicates *Z* < 1, black indicates *Z* > 2, and the grayscale varies with Z between 1 and 2.

The overall validity of the model was determined by *Z*‐score analysis. Each measured experimental endpoint was simulated, and the simulated value was given a *z*‐score based on the experimental distribution. The outcome of this study is shown in Figure 8. The model predictions lay more than two standard deviations from the experimental results 5 of 161 times, and between one and two standard deviations from the experimental mean 19 of 161 times. Additionally, root mean square errors are shown for each protocol and variable as a measure of the deviation of the error of the simulation as compared to the particular observation and protocol.

## Discussion

Water homeostasis is one of the body's most critical tasks. Physical challenges to the body, including exercise and surgery, almost always coordinate with some change in water handling reflecting the changing needs of the body. Maintenance of water homeostasis in a healthy individual is a matter of multiple control systems that interact directly and indirectly. The partition of extracellular water into interstitial and vascular compartments, the actions of the kidneys to rid the body of excess fluids and salt, and the renal hormones, both vasoactive and inactive, combine to form a complex network of forces competing to accomplish similar and parallel goals: the preservation or diminution of the body's sodium and water loads.

In this work, we tested the validity of HumMod, an integrative physiological mathematical model, in a variety of challenges to water homeostasis, emphasizing its performance in simulating changes in serum osmolarity, serum sodium concentration, and serum AVP. This effort is not intended to describe the method of parameterization of the model, which was done by on a relationship‐by‐relationship basis to best fit experimental data, mostly from animals. We present the model's responses to under‐ and over‐hydration, and recovery from these states via a variety of strategies. The protocols we used replicate human experiments, and we transcribed data from a small collection of papers to demonstrate HumMod's validity. We only concentrated on dehydration through water restriction and through hypertonic infusion and not an exercise protocol in order to minimize the confounding effects of metabolic and sympathetic disturbances. The original results for the experimental studies were reported as mean and standard error; we converted these to standard deviations to allow an intuitive z‐score analysis. As well, we present the root mean square error of each variable observed in the protocols to numerically quantify model error. Although the model could more closely replicate these protocols, changing model parameters would result in a rejection of the relation‐level calibration, and would nullify any claims made as to the validity of the model.

Most mathematical models simulate a single process or small network of processes, trading breadth for accuracy with respect to a small number of studies. HumMod is designed to be a different type of model, encompassing multiple control systems, each built originally as a small model but sewn together to create a multisystem model. The purpose of HumMod is to simulate as many physiological actions as possible in a computationally efficient framework. We sacrifice many complexities of physiology for this purpose: pulse waves, local tissue effects at the microscopic level, and action potentials are all ignored in our model. Instead, we reduce the human body to organ or tissue level (e.g., *α*,* β*,* γ,* and *δ* cells in the pancreas) to allow system‐level interactions to develop, similar to the approach of Guyton and Coleman (Guyton et al. 1972). This allows more complex validation to take place. Rather than considering the model response to a single type of challenge, or to a single system, the model can be tested simultaneously in multiple ways, adding confidence that the simulation outputs reflect reality. In our case, the three types of prefix protocols: restriction hypernatremia, salt loading hypernatremia, and normal state, are combined with four types of suffix protocols: water restriction, *ad libidum* water, water loading, and saline loading. These protocols offer challenges to salt retention, water partition, and water retention. In the studies we used to validate our model, plasma renin activity was observed but did not significantly change. HumMod behaved similarly, so those results were not included. Significant changes in AVP, serum osmolarity, and serum sodium were observed in multiple studies, so we focused our exposition on these factors.

In all cases, HumMod was able to match the overall changes in serum osmolarity, AVP, urine osmolarity, hematocrit, serum protein concentration, ANP, and serum sodium concentration, only falling outside the first standard deviation 23 times, and outside of the second standard deviation 4 times, over 161 total observations. Moreover, the AVP‐osmolarity serum curves were very similar to those extracted from two different published experimental protocols. Recalling that the secretion mechanism for AVP is also influenced by nervous reflexes associated with pressure, we believe that this demonstrates a successful integration of multiple systems.

The most integrated response we observed, however, is cumulative water intake (CWI) following induced hypernatremia with normovolemia (Protocol E). This response integrates serum sodium and osmolarity with AVP, the thirst mechanisms, and the gastrointestinal tract's absorption of water. Taken together, the closely fitted responses of CWI, AVP, sodium concentration, and serum osmolarity indicate that our model captures all of the important apparent features of the human response to acute hypernatremia, which has not been successfully replicated before.

The most challenging aspect of AVP secretion to quantify experimentally is the interaction of neural stimulus and osmolarity. Only two of the protocols we considered, Thompson's hypervolemic hypernatremia (E and F) measured pressure, and no significant differences were observed throughout the protocol in either the human experiments or the model. The model did not predict significant changes to mean arterial pressure in the other protocols either, so it is possible we were able to restrict our attention to only the osmolarity stimulus, the thirst mechanism, the partition of body water, and the actions of the kidney toward fluid maintenance.

One limitation of the model was in predicting urine osmolarity after an acute water load in Protocol A. Goldsmith did not report data other than urine osmolarity, which makes comparison difficult. The significant increase in water excretion matches the rates of 20 L of water intake observed at balance in human patients with diabetes insipidus (DI), which suggests that salt retention, or lack thereof, may be the source of the discrepancy. While the model demonstrated a 33% increase in ANP after acute water load, one source noted no significant change in ANP in a similar study (Burrell et al. 1991).

In conclusion, HumMod provides a realistic simulation of the selected protocols challenging interstitial fluid balance, intracellular/extracellular fluid balance, AVP secretion and action, and thirst. We believe that HumMod demonstrates sufficient similarity to reported responses to salt/water homeostasis to serve as a basis for future investigations.

## Conflict of Interest

None declared.

## Figures and Tables

**Table A1:**
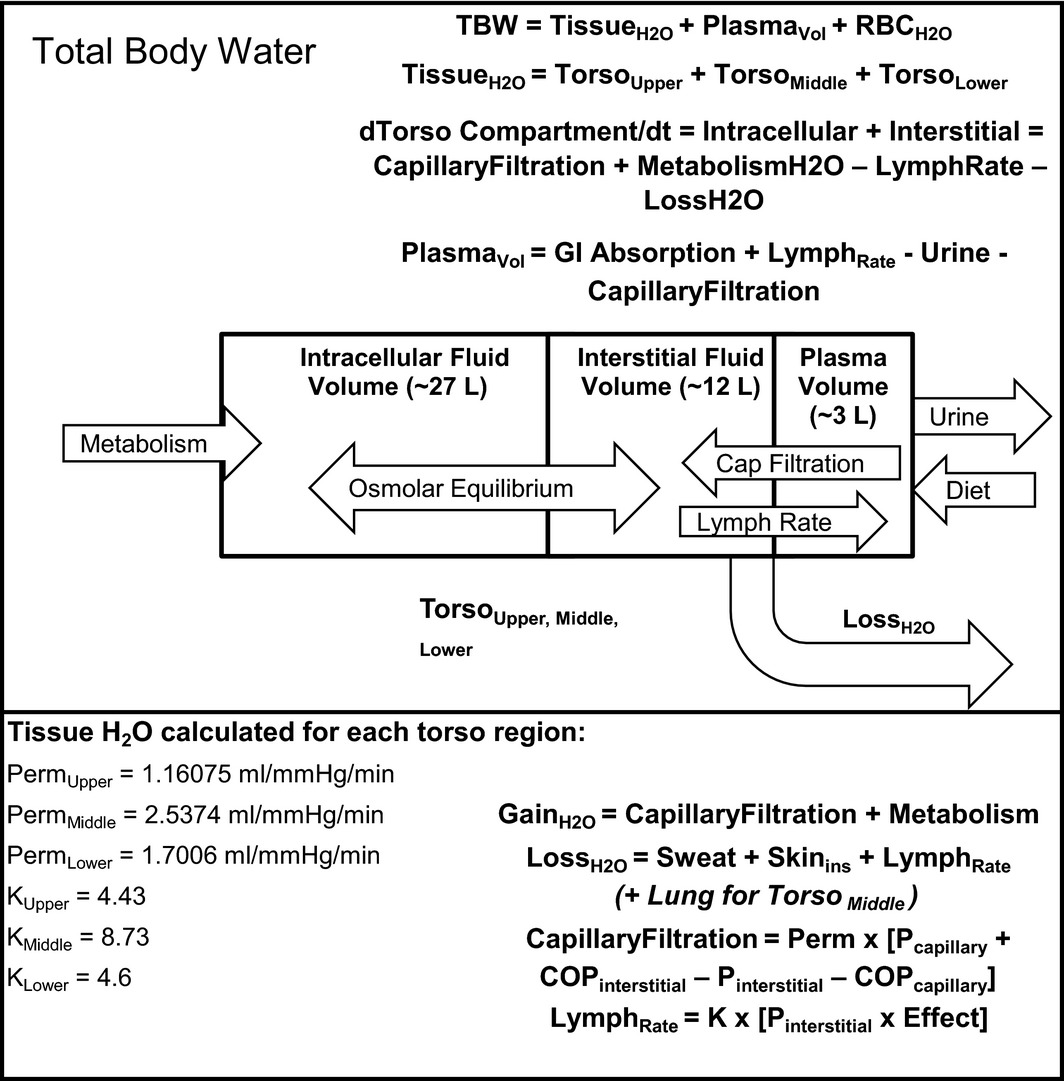
Volume and water transport regulation. Total body water is calculated by the sum of three regions (torso compartments), each of which is divided into intracellular and interstitial spaces. Important calculation, variables, parameters are listed. Also included is a diagram of the water flux between the total intracellular, interstitial, and plasma fluid spaces with approximate volumes

**Table A2:**
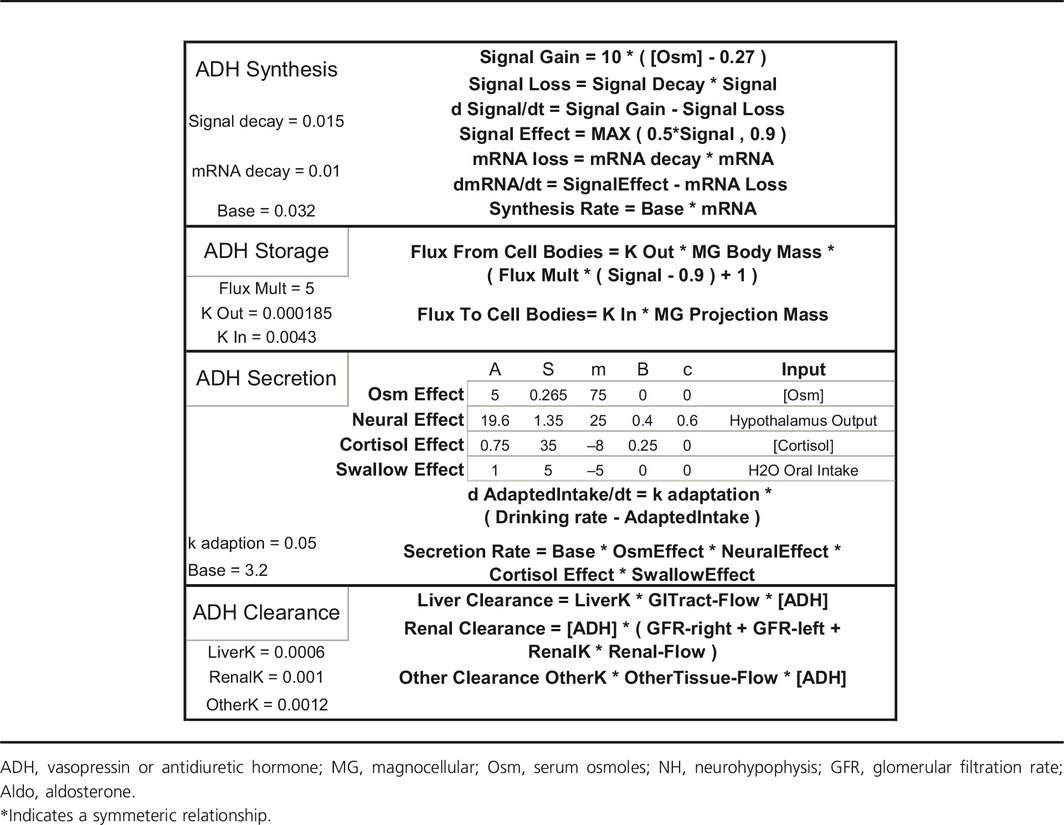
ADH synthesis and secretion. The dependent variables are displayed in the left column, whereas factors and effects that impact the dependent variables are listed in the middle column, along with their effect range and input variable that determines the effect. Calculations are provided for some variables

**Table A3:**
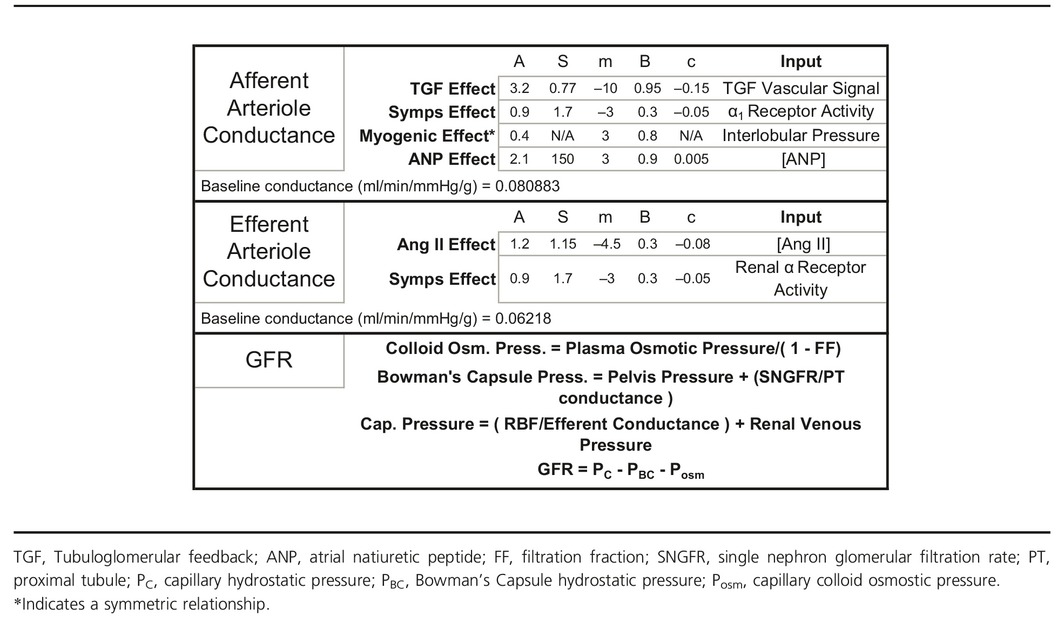
Control of renal vascular resistance and GFR. The dependent variables are displayed in the left column, whereas factors and effects that impact the dependent variables are listed in the middle column, along with their effect range and input variable that determines the effect

**Table A4:**
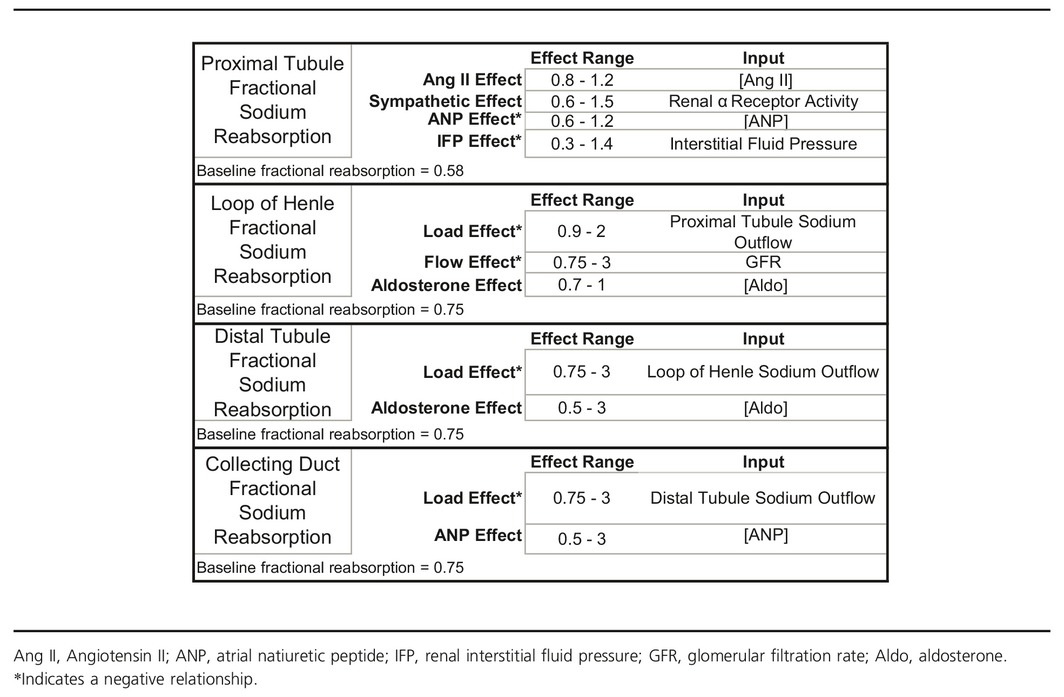
Control of sodium reabsorption in the nephron. The dependent variables are displayed in the left column, whereas factors and effects that impact the dependent variables are listed in the middle column, along with their effect range and input variable that determines the effect

**Table A5:**
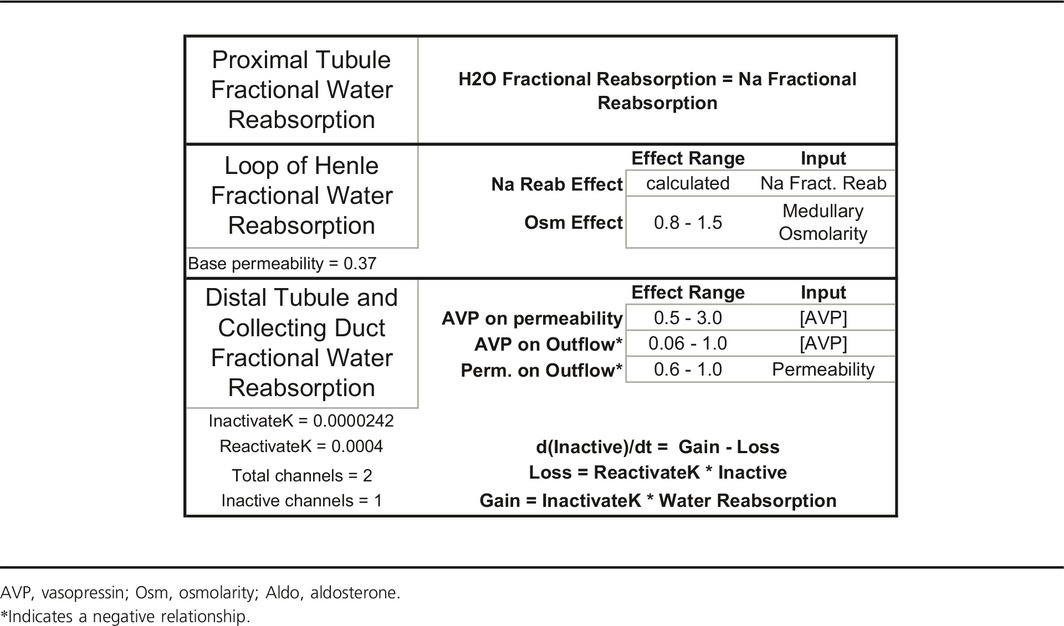
Control of water reabsorption in the nephron. The dependent variables are displayed in the left column, whereas factors and effects that impact the dependent variables are listed in the middle column, along with their effect range and input variable that determines the effect. Calculations are provided for some variables

**Table A6:**
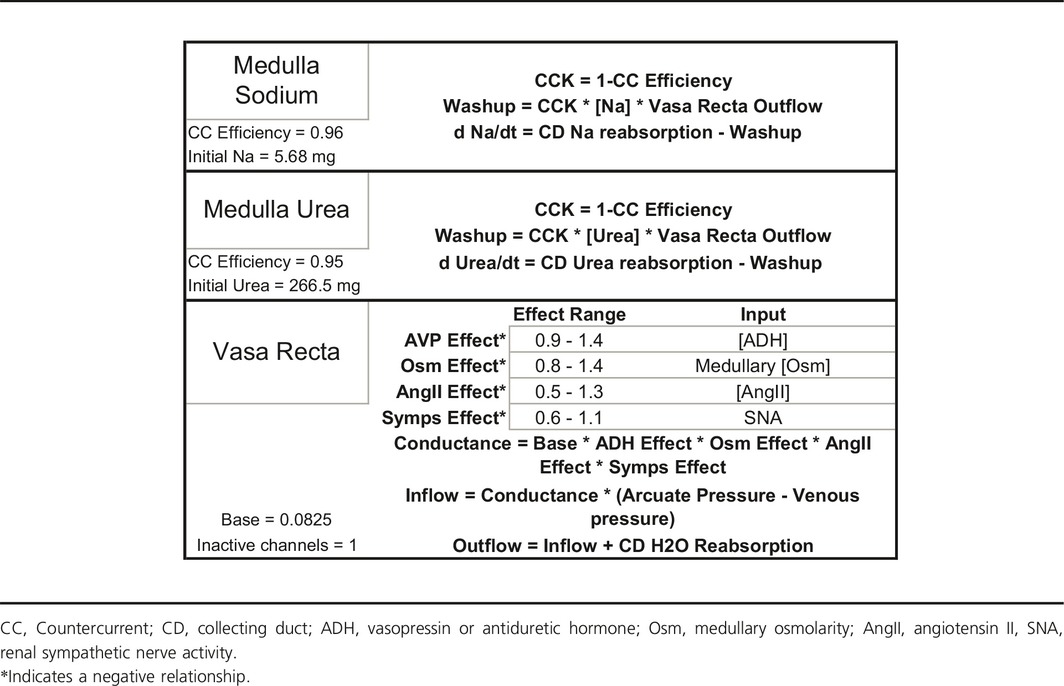
Rcts that impact the dependent variables are listed in the middle column, along with their effect range and input variable that determines the effect. Calculations are provided for some variables

**Table A7:**
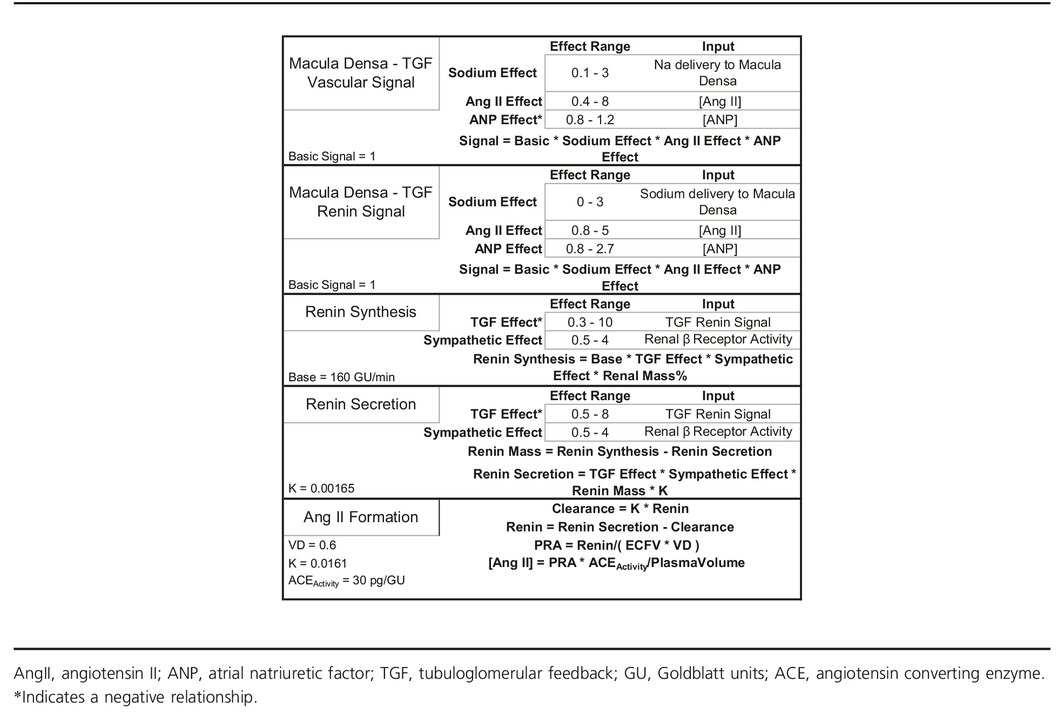
Regulation of tubuloglomerular feedback (TGF) and renal hormones. The dependent variables are displayed in the left column, whereas factors and effects that impact the dependent variables are listed in the middle column, along with their effect range and input variable that determines the effect. Calculations are provided for some variables where indicated

**Table A8:**
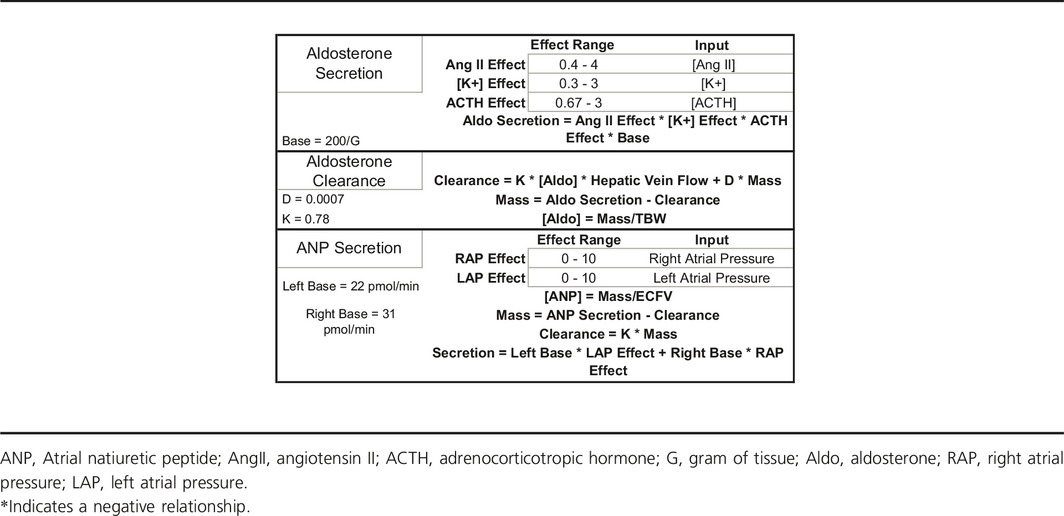
Regulation of relevant nonrenal hormones. The dependent variables are displayed in the left column, whereas factors and effects that impact the dependent variables are listed in the middle column, along with their effect range and input variable that determines the effect. Calculations are provided for some variables where indicated

**Table A9:**
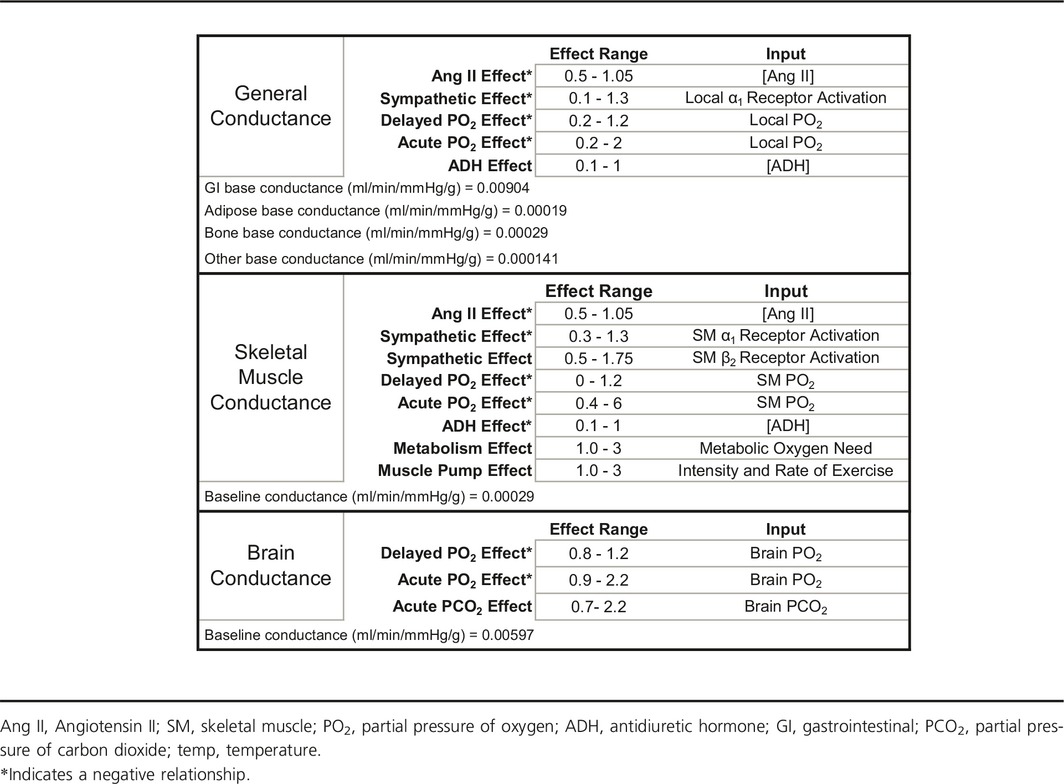
Control of organ vascular conductance. The dependent variables are displayed in the left column, whereas factors and effects that impact the dependent variables are listed in the middle column, along with their effect range and input variable that determines the effect. Conductance is calculated from the product of the effects and the baseline conductance. “Local” tissues are the GI tract, Bone, Adipose tissue, and nonspecific other tissue. Skin flow, with its dependence on temperature, is not shown here

## Supplementary Model Description

### Curve Fitting

The individual responses of hormones in the model were fit from human, dog, swine, or rat data, with a preference toward human data. Observing the integration of the individual responses to controlled stimuli in more complicated, uncontrolled situations is the chief goal of this model. For this reason, controlled experiments in humans or animals were used to characterize the individual relationships between hormones and their secretagogues. In the kidney, ex vivo preparations, chiefly from animal experiments, but with some human tissue obtained during the course of standard medical procedures such as tumor resection, were used to parameterize the relationships shown. In some cases, these parameterizations can be confirmed or augmented with the use of particular agents such as lithium, which correlates with proximal tubule water and sodium reabsorption (Koomans et al. 1989). Sigmoids are all fit as Hill equations, either solved as unique solutions in the context of limited data, or with least square methods where sufficient input points could be determined from the literature.

### Volume and Water Homeostasis

Water enters the system through food and drink, metabolism, and intravenous addition, and leaves the system via urine, feces, sweat, and insensible loss through skin and lungs. HumMod contains three major water compartments: the intracellular, interstitial, and plasma pools. Electrolytes are evenly distributed between the plasma and interstitial pools, so the regulation of their relative volumes is guided by fluid pressure and oncotic pressure in the respective compartments. Water leaves the vasculature through capillary filtration, and is returned to the vasculature through lymph flow. Water is able to quickly pass between intracellular and extracellular (summed plasma and interstitial) volumes. The relative volumes of these compartments are governed by osmolality. A second compartmentalization is given by dividing the body into upper, middle, and lower torso compartments. These compartments allow simulation of orthostasis, but do not play a role in the current work. See Table A1.

### AVP Model

#### Hypothalamic‐pituitary model

AVP is synthesized in the magnocellular nerve bodies and transported to the pituitary for release. In rat experiments, enough peptide for 10 days of normal secretion are present, and we have made the assumption that a similar amount is stored in humans. This is supported by the observation that DI only becomes apparent 7 days after the infundibulum is damaged in transsphenoidal surgeries (Olson et al. 1997). It has been shown that transcription rate is the primary regulator of synthesis, and that mRNA is decreased when the amount of stored AVP is high (Fitzsimmons et al. 1994). Because no protocol exceeded 42 h, synthesis and transport of AVP were not important in the model response, and no further description will be made. We do note that base secretion is 3.2 ng/min (Lauson 1951; Robinson et al. 1989).

Secretion is driven by two factors, serum osmolarity and direct nervous stimulation. We use a black box model to generate sigmoidal multipliers for both factors. The osmolarity model is fit from data reported by Robertson (Robertson et al. 1973). We use only serum osmolarity as the input for this function. The neural stimulation model is fit from data observed in normal and heart‐transplanted humans, supplemented with conscious canine data (Giannattasio et al. 1991; Thrasher et al. 2000). The neural stimulus originates at the carotid and cardiac baroreceptors and is integrated in the paraventricular nucleus (Segar and Moore 1968; Giannattasio et al. 1991). We consider the low pressure receptors to be quantitatively more important than the arterial baroreceptors (Giannattasio et al. 1991). Additionally, it has been noted that swallowing generates an oropharyngeal reflex that curtails secretion temporarily (Burrell et al. 1991). This reflex is modeled with a graded switch *Sallow*, which takes adapted per minute water intake as its argument. Adaptation takes place with a time constant of 5 min.

These effects are combined to generate total secretion. Some early work indicated that autonomic blocking agents were able to completely eliminate the AVP response to high serum osmolarity (Bridges and Thorn 1970), leading us to considering the interactions as multiplicative.

#### Pharmacodynamic model

We set the volume of distribution of AVP to be the full ECFV in line with previous reports (Benmansour et al. 1982).

AVP is cleared in the liver, in the kidney, and elsewhere (Lauson et al. 1965; Lauson 1967). There is little agreement concerning the precise balance of extraction in the various tissues. In a study of human patients beginning dialysis, plasma clearance rate was 50% lower than in healthy controls (Pruszczynski et al. 1984; Argent et al. 1992) suggesting that the kidney is responsible for about half of all clearance. A study in healthy humans with radioiodinated AVP reported renal clearance of 80 mL/min (half‐time 24 min), with 27% of total due to the kidney (Baumann and Dingman 1976). Previous work has demonstrated that I_125_‐AVP has a slower total clearance from circulation with a half‐time of 28 min, indicating that the human study may be underestimating renal clearance. Dogs are more heavily studied, but differ from humans in the following ways. In dogs, urinary clearance is 70–100% of GFR and whole organ clearance is 20–30% greater (Lauson et al. 1965; Share et al. 1985). In addition, canine renal clearance is about 27% of total clearance (Matsui et al. 1983). These two data are consistent with one another, and suggest that in the kidney, AVP is filtered with no reabsorption and is also cleared via a non‐tubular path. We suggest a renal reabsorption model comprised of 100% clearance of filtered plasma and fractional clearance of the remainder. Splanchnic and “other” clearance are handled as fractions of splanchnic flow and fraction of plasma concentration.

We set *k*_renal_ = 0.001, and *k*_hep_ = 0.0006. Together, these give steady state clearances of 1331 pg/min and 727 pg/min, respectively, with a ratio of 23% hepatic and 43% renal. This agrees with the relative ratio of renal to hepatic clearance of 2 as reported by Lauson (Lauson et al. 1965). These numbers do not critically affect this model as we assume the individuals are primarily resting during their observation period so renal and hepatic blood flows are left near baseline. GFR, RPF (renal plasma flow), and HPF (hepatic plasma flow) (all in mL) are calculated by HumMod as functions of other systemic factors. The sum of these clearance yields the total clearance of AVP.

#### Parameterization

To parameterize the model, steady state assumptions are used. The concentration of AVP in the rat pituitary was measured as 259 mU/100 g (Jones and Pickering 1969). In dogs, the axonal projections are believed to contain 10–20% of total AVP stores (Sachs et al. 1967). This was supported by observations that the pituitary content of AVP is sufficient to maintain normal secretion amounts for 30–50 days, or maximal secretion amounts for 5–7 days (Robinson and Fitzsimmons 1993). These data, along with metabolic clearance rates of circulating AVP, were assimilated to construct the amounts of AVP in the axonal and hypothalamic parts of the AVP containing neurons. The readily available pool constitutes 10–20% of total supply (Sachs et al. 1967). Given average circulating levels and known clearance characteristics, we can set the initial volume of AVP in the cell projections as Mass_P_ = 17000 ng, and the initial mass in the hypothalamus as Mass_H_ = 173000 ng. The AVP model is described in Table A2.

### Renal Model

#### Glomerular filtration

Glomerular filtration is calculated by considering the relative fluid and oncotic pressures in the glomerular capillaries and Bowman's capsule. The glomerular capillary pressure is profoundly affected by afferent and efferent arteriolar conditions, notably their conductance and relative pressures. These are controlled by tubuloglomerular feedback (TGF) and angiotensin II (AII) (Edwards 1983), respectively, and by *α*_1_‐adrenergic activity in both zones (Zimmerman et al. 1984; Kon 1989). TGF and AII are described in the sequel. The GFR model is summarized in Table A3.

#### Sodium reabsorption

Because of its correlation with water absorption especially in the proximal half of the nephron, we describe sodium reabsorption. HumMod contains four distinct tubule zones: the proximal (PT), loop of Henle (LH), distal tubule (DT), and collecting duct (CD). In the proximal tubule, sodium reabsorption is driven by sympathetic activation, AII, and ANP (Berl et al. 1974; Olsen et al. 1985; Firth et al. 1988; Koomans et al. 1989; DiBona and Kopp 1997). In many places, interstitial fluid pressure has also been implicated in driving reabsorption by solvent drag (Engeli and Sharma 2001). In the loop, aldosterone and the transport velocity of active sodium transporters comprise the chief modulators of reabsorption (Stumpe et al. 1971). In the distal tubule, active transport velocity and aldosterone modulate transport magnitude, and in the collecting duct, ANP increases sodium reabsorption. The sodium reabsorption model is summarized in Table A4.

#### Water reabsorption

Water is reabsorbed along with sodium through the nephron with some modulation in the distal nephron by hormonal factors. In PT, water moves with sodium in the same fractional rate (Schrier et al. 1975). In LH, the osmolarity of the medulla drives water reabsorption as a multiple of sodium fractional reabsorption. In the distal tubule and collecting ducts, the intercalated cells, increased numbers of Aquaporin‐2 (AQP2) water channels increase the permeability of the distal tubule or collecting duct to water. ACP2 synthesis is stimulated by AVP (DiGiovanni et al. 1994), and AVP binding to AVPR2 causes AQP2 to move to the basolateral surface of tubule cells. Hence, AVP increases the numbers of active (basolaterally expressed) and inactive (cytosolic) AQP2 (Boone and Deen 2008). For simplicity, we assume that both pools are affected equally by AVP, and that the effects track changes in AVP levels instantly. The water reabsorption models are summarized in Table A5.

#### Renal medulla

The medulla impacts reabsorption of water in the PT, DT, and CD. Two molecules, sodium and urea, are simulated in the renal medulla model. Both sodium and urea are added to the medulla from the CD and are washed out with vasa recta flow. The washout is modulated by countercurrent efficiency. The vasa recta flow is subject to adjustment by AVP, AII, sympathetic nerve outflow, and by medullary osmolarity (Schmid‐Schonbein et al. 1973; Cupples et al. 1988; Turner and Pallone 1997; Crawford et al. 2013). As the renal medullary concentration of urea and sodium increase, water is drawn from the vasa recta into the interstitium (Morgan and Berliner 1968). The renal medulla model is summarized in Table A6.

### Hormones

#### Renal hormones

Renin, and its enzymatic product angiotensin II, are the chief hormonal output of the kidney. Renin is secreted from the macula densa, a sensory organ between LH and DT, adjacent to the afferent arterioles. In HumMod, the specialized cells of the macula densa sense sodium concentration in transit to the distal portions of the tubule and adjust renin secretion and afferent arteriolar conductance to stabilize sodium delivery to the distal tubule (Oelkers et al. 1978). Renin synthesis and secretion are controlled by TGF and by sympathetic stimulation (Bunag et al. 1966; Riegger and Liebau 1982). AII is formed enzymatically by renin and the activity of angiotensin converting enzyme and acts to promote sodium reabsorption, constrict peripheral vessels, and sensitize TGF (Ploth and Roy 1982; Olsen et al. 1985). The renal hormone models are described in Table A7.

#### Other hormones

Aldosterone and atrial natriuretic peptide (ANP) also play a role in HumMod's water homeostasis. Aldosterone, produced by the adrenal glands is stimulated by AII, potassium, and ACTH (Aguilera and Catt 1978). Aldosterone acts in the loop of Henle and distal tubule to promote sodium reabsorption, and in the distal tubule to enhance potassium secretion. ANP is synthesized in the left and right atria. Secretion is driven by increases in atrial pressure. ANP acts in the PT and CD to inhibit sodium reabsorption. These hormones are summarized in Table A8.

### Vascular Model

AVP is a potent vasoconstrictor. Systemically, AVP infusion results in increased peripheral resistance (Heyndrickx et al. 1976). Similar decreases in conductance in the renal vasculature and iliac bed have been reported (Heyndrickx et al. 1976). In pigs, conductance was decreased by 29%, 18%, 23%, and 34% in brain, heart, left and right kidney, respectively (Muller et al. 2008). Only the hepatic artery is seemingly immune to its vasoconstrictive effects: conductance increased by 25% in cats (Krarup 1975) and has, in general, been noted to oppose changes in portal vein flow, possibly by an adenosine related mechanism (Lautt and Legare 1986). Other factors that alter hemodynamics in tissue beds are shown for completeness (Green and Kepchar 1959) and summarized in Table A9.
